# Editorial: Uncertainty, Anxiety, and Fear of Cancer Recurrence

**DOI:** 10.3389/fpsyg.2021.811602

**Published:** 2021-12-10

**Authors:** Phyllis Butow, Sophie Lebel, Joanne Shaw, Gerry Humphris

**Affiliations:** ^1^The University of Sydney, Darlington, WA, Australia; ^2^Faculty of Science, School of Psychology, The University of Sydney, Sydney, NSW, Australia; ^3^School of Psychology, University of Ottawa, Ottawa, ON, Canada; ^4^School of Medicine, University of St Andrews, St. Andrews, United Kingdom

**Keywords:** uncertainty, anxiety, fear of recurrence, editorial, cancer

In this topic, we sought to bring together the related topics of uncertainty, anxiety, and fear of cancer recurrence or progression (FCR). As these responses are common, can be severe, impact treatment decision making, and impact quality of life (Simard et al., 2013; Shim et al., 2018), collectively they represent key concerns for psycho-oncology.

Uncertainty is a patients' inability to determine the meaning of illness-related events, and can be a result of ambiguity (conflicting, incomplete or inadequate information); complexity (information that is difficult to understand); and unpredictability (likelihood or risk of the future outcome of the disease) (Mishel, 1988). Anxiety in cancer can arise due to existential threat, uncertainty, fear of uncomfortable tests, treatments and side-effects, and loss of meaning and coherence. Meanwhile, FCR is the fear, worry or concern relating to the possibility that cancer will come back or progress, and may relate to fear of death and dying, fear of undergoing aversive treatments again, or other issues (Lebel et al., 2016). Impacts of these constructs have been described as behavioral (e.g., increased or decreased surveillance and body-checking), cognitive (e.g., intrusive thoughts of cancer), emotional (e.g., distress), and social (e.g., inability to plan holidays for fear of a recurrence) (Lebel et al., 2016).

As Editors, we were delighted by the response to this topic, and believe this collection of papers provides the most comprehensive overview of current research in this area in the literature. The universal nature of these concerns is highlighted through contributions from researchers all around the world, including Canada, US, Australia, Europe, UK, Hong Kong and China. Topics covered are wide-ranging. New researchers to the field will find this a useful body of work with which to start familiarizing themselves with the current studies and groups globally.

Overall, we have 25 accepted papers. Two papers provided a review of the conceptualization of these or related concepts, highlighting the need to thoughtfully consider what we mean when we use terms, to define them carefully and to continue efforts to clearly articulate their similarities and differences. Maheu et al. reviewed conceptualisations of FCR, health anxiety, worry and illness uncertainty. They found all concepts were triggered by internal somatic and external cues, but that each had unique aspects also. Overall, they concluded that FCR and illness uncertainty were more likely to be triggered by cancer-specific factors, while worry and health anxiety were more trait-like. Kühne et al. reviewed conceptualisations of prognostic awareness. These authors highlighted the different aspects included under this term (such as knowledge of the chances of recovery, acknowledgment of a limited lifespan, an accurate life expectancy and knowledge of therapy goals).

Five papers reported results of systematic reviews. Anderson et al. reviewed the literature on FCR in indigenous and minority groups, identifying 19 articles. They identified some differences in the severity and correlates of FCR between cultural groups, albeit most being inconsistent. Importantly, their findings highlighted the need to consider cultural factors when assessing and treating FCR. O'Rourke et al. examined the small literature on factors associated with FCR in caregivers, noting relationships between age, treatment modality and illness perceptions and caregiver FCR. Stewart et al. reviewed the impact of cancer type on patients' experience of a cancer recurrence; however relevant papers addressed only breast and prostate cancer, limiting conclusions. Notably Naser et al. in a Middle Eastern sample found higher rates of depression in bladder cancer patients and anxiety in lung cancer patients. Pang and Humphris completed a review and meta-analysis of data examining the association between FCR and gender, firmly concluding that females have higher FCR than males. However, these authors noted only moderate effect sizes, suggesting that other factors are more key in determining FCR levels.

Finally, Williams et al. conducted a timely review of the cost of delivering FCR interventions. This is a critical and only newly emerging field of enquiry, which is essential if health care systems and decision makers are to be convinced of the value of funding FCR intervention programs. The review concluded that FCR is associated with greater use of healthcare resources, and can be treated cost-effectively, although additional measures and approaches are needed in future studies.

Four articles explored FCR and existential distress in novel contexts. Soriano et al. explored the impact of treatment delays caused by the COVID-19 pandemic on FCR. Women with ductal carcinoma *in situ*, lobular carcinoma *in situ*, or invasive breast cancer, whose cancer surgery was postponed due to the pandemic, completed questionnaires while they awaited their surgery or shortly after they received their delayed surgery. Reassuringly these women reported low psychosocial impacts, although as the authors noted, FCR often emerges during follow-up, thus longitudinal studies will be required to really assess the impact of COVID-19 on psychosocial cancer outcomes.

Rogers et al. focused on FCR in head and neck patients, noting that young women are particularly vulnerable to FCR and may require specialist attention. Custers et al. explored FCR longitudinally in women treated curatively with breast cancer. While a number of longitudinal studies have now been published, they still represent a small proportion of the literature. Custers et al. study highlights the often-fluctuating nature of FCR over time, and thus the importance of assessing FCR on multiple occasions in order not to miss significant morbidity and need for help. Finally, Sobota and Ozakinci explored a topic that has previously been largely ignored: fertility concerns and fear of cancer progression (FOP) in a vulnerable population, young women. This paper highlighted how FCR and FOP may be weighed up against other priorities when people make treatment decisions impacting diverse outcomes.

Two studies sought to further the growing literature on cognitive biases in FCR using experimental paradigms (dot probe tasks). Ng et al. noted attentional bias away from threat and a negative interpretation bias in women with persistent distress after breast cancer, suggesting that attention focus training, reducing threat salience or modifying threat appraisal may help this group. Similarly, Tuman et al. reported higher threat endorsement was linked to higher overall fear and mediated the relationship between experiencing somatic symptoms and FCR. As therapy which includes attention to cognitive biases has been shown to be particularly effective for FCR (Tauber et al., 2019), further attempts to understand their role, and how best to modify them, is needed.

Four papers explored personality factors associated with FCR and distress, including attachment anxiety (Graf et al.), extroversion (Alvisi et al.), and daily and pathological worry (Dinkel et al.), while Seguin Leclair et al. noted that illness beliefs and health self-efficacy can impact FCR. While some of these factors are not readily modifiable, they may represent vulnerability factors to which clinicians can be alert.

Five papers specifically explored uncertainty in cancer patients. Han et al. reported results of a qualitative study in women with ovarian cancer, noting that patients cope with, construct and maintain uncertainty in an ongoing effort to maintain hope. Bartley et al. noted a desire to reduce uncertainty in their sample of patients undergoing whole genome sequencing; patients with greater uncertainty after testing reported higher anxiety at a 12-month follow-up. Similarly, Reyes et al. explored experience of uncertainty in people with a pathogenic or likely pathogenic variant in ATM or CHEK-2, both moderate-risk cancer genes. They found that such ambiguous data was a major source of uncertainty to participants, with the potential to impact subsequent uptake of cancer risk management recommendations. These studies suggest that uncertainty is an issue that should be explored with cancer patients over time, as patients grapple with the uncertainties and realities of their disease, including the relatively new uncertainties provided by genetic and genomic results.

van Someren et al. paper nicely complements this work, by exploring how oncologists address uncertainty in audiotaped consultations. They identified seven different approaches, including explaining the reasons for and degree of uncertainty, and down-playing uncertainty. In a similar analysis of audiotaped oncology consultations with patients who have advanced cancer, Larsen et al. explored how patients expressed existential distress. They detected tentative, controlled and often indirect expressions of uncertainty about the future, uncertainty about own coping, and search for meaning. These findings emphasize the vulnerability and fear of patients in this situation, and the need for oncologists to skillfully explore patients' concerns and provide information and support where possible to address them. While not directly focusing on uncertainty, van Beusekom et al. report the co-design of communication skills training for radiologists to address distress in cancer patients, captured by the acronym KEW (Know, Encourage, Warmth).

Stepped care is emerging as a key strategy to increase access to cost-effective support and treatment for FCR (Cancer Australia, 2013). While no paper addressed this directly, three papers evaluated interventions which could complement face-to-face intensive therapy for FCR and distress. Pradhan et al. evaluated a simple online FCR booklet for women with ovarian cancer addressing FCR. While acceptable, it proved ineffective in improving FCR. Kan et al. provided acceptability data on a phone-delivered therapy supplemented by a booklet for people with high -risk melanoma. This proved highly acceptable and was particularly effective for those with higher FCR at baseline (who perhaps had more need). Finally Zhou et al. evaluated group-delivered reminiscence therapy for cancer patients, demonstrating impact on anxiety and depression.

As this field develops, it is increasingly clear that suitable interventions are needed for different levels of FCR to ensure timely and universal access and sustainability. Further, we need to maintain a high standard of rigor in our intervention research, with attention to process as well as outcomes. To this end, Brandt et al. in this topic report on a useful fidelity tool with which to measure the extent that a manualised intervention is delivered according to instructions. Without such assessment, it is not possible to determine whether interventions are effective (or ineffective) due to the therapy content or because novel elements are introduced, or planned ones omitted.

Finally, Shaw et al. reported findings from an international Delphi study examining priorities for research in FCR moving forward. Intervention research, strategies to increase patient access to FCR treatment, evaluation in real world settings and continuing to define mechanisms of action and active components of interventions, were highlighted. These priorities nicely reflect the body of work included in this topic and suggest that the research community is working collaboratively and coherently on these issues.

A further issue cannot escape comment. Without warning, from the beginning of 2020, the world of health care has been transformed in outlook, in its delivery and response from staff and services. The impact of the COVID-19 pandemic has been an undeniable feature that threatens further to increase uncertainty, raise anxiety and possibly fears of cancer recurrence or progression. New knowledge about transmission and new variants of the virus, the likely chronic effects of long COVID and the known benefit of immunization introduces new elements of complexity. For example, long COVID has an array of presentations that may add to confusion for patients and clinical teams in identifying possible new tumors, and increase the variability of experiences of uncertainty and fears. The call for papers for this special issue did result, as mentioned previously, in a single manuscript that reported the consequence of delay due to COVID-19 on fear of recurrence. Researchers are unable to ignore the relative effects of the pandemic and will need to encompass the new working conditions for staff and patient experience in their studies to prepare bids for future resources and research activity.

In conclusion, there is much to investigate regarding anxiety, uncertainty and fear of recurrence in the context of cancer and more broadly in other diseases. We need more fruitful research to guide our understanding of the development and outcomes of these responses, how they interact, and how best to help people manage and minimize their impact on quality of life and well-being. These constructs need examination in older adult, adolescent and young adult and pediatric settings; most research to date has been in adult populations. This will require more researchers attracting funding and interest in this topic. As an international community, we need to work together to achieve these goals.

## Author Contributions

All authors contributed to the article and approved the submitted version.

## Conflict of Interest

The authors declare that the research was conducted in the absence of any commercial or financial relationships that could be construed as a potential conflict of interest.

## Publisher's Note

All claims expressed in this article are solely those of the authors and do not necessarily represent those of their affiliated organizations, or those of the publisher, the editors and the reviewers. Any product that may be evaluated in this article, or claim that may be made by its manufacturer, is not guaranteed or endorsed by the publisher.
